# Investigating Biomolecules in Deep Eutectic Solvents with Molecular Dynamics Simulations: Current State, Challenges and Future Perspectives

**DOI:** 10.3390/molecules29030703

**Published:** 2024-02-02

**Authors:** Jan Philipp Bittner, Irina Smirnova, Sven Jakobtorweihen

**Affiliations:** 1 Institute of Thermal Separation Processes, Hamburg University of Technology, Eißendorfer Straße 38, 21073 Hamburg, Germany; 2 Institute of Chemical Reaction Engineering, Hamburg University of Technology, Eißendorfer Straße 38, 21073 Hamburg, Germany

**Keywords:** review, biomolecules, enzymes, proteins, deep eutectic solvents, molecular dynamics simulations, protein–DESs interactions

## Abstract

Deep eutectic solvents (DESs) have recently gained increased attention for their potential in biotechnological applications. DESs are binary mixtures often consisting of a hydrogen bond acceptor and a hydrogen bond donor, which allows for tailoring their properties for particular applications. If produced from sustainable resources, they can provide a greener alternative to many traditional organic solvents for usage in various applications (e.g., as reaction environment, crystallization agent, or storage medium). To navigate this large design space, it is crucial to comprehend the behavior of biomolecules (e.g., enzymes, proteins, cofactors, and DNA) in DESs and the impact of their individual components. Molecular dynamics (MD) simulations offer a powerful tool for understanding thermodynamic and transport processes at the atomic level and offer insights into their fundamental phenomena, which may not be accessible through experiments. While the experimental investigation of DESs for various biotechnological applications is well progressed, a thorough investigation of biomolecules in DESs via MD simulations has only gained popularity in recent years. Within this work, we aim to provide an overview of the current state of modeling biomolecules with MD simulations in DESs and discuss future directions with a focus for optimizing the molecular simulations and increasing our fundamental knowledge.

## 1. Introduction

As the catalyst of nature, enzymes have become increasingly important for industrial applications as they can catalyze many relevant reactions with unmatched reactivity and selectivity. The selection of the solvent is thereby a crucial factor for optimizing (bio)chemical processes to meet the current and future needs for sustainable industry. As a greener alternative to many classical organic solvents, deep eutectic solvents (DES) have emerged over the past decade as a new solvent class for (bio)chemical applications. They have even been framed as the ‘solvent of the 21st century’ [1].

### 1.1. Definition of Deep Eutectic Solvents

**Figure 1:**
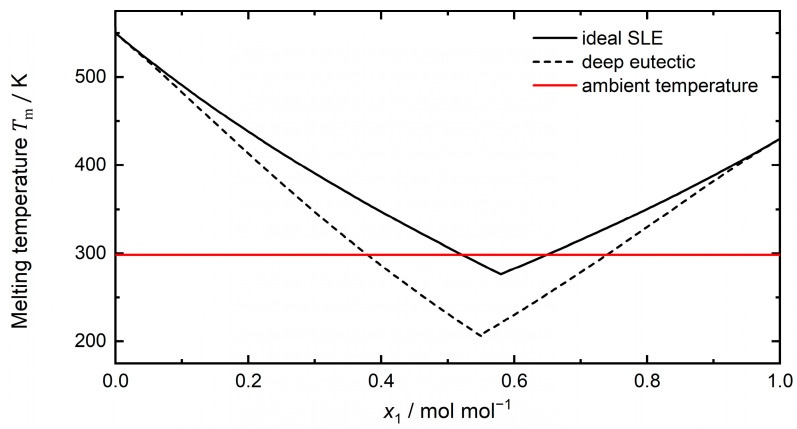
Phase diagram (solid–liquid equilibrium) of an arbitrary eutectic system for the ideal eutectic (solid black line) and a deep eutectic (dashed black line) in dependency of mole fraction of component 1 (*x*_1_). This figure shows an arbitrary negative deviation from ideality as the deep eutectic, while different forms of deep eutectics exist [3]. The ambient temperature (red solid line) is added as reference.

Firstly reported by Abbott et al. [2] in 2003, DESs are often described as binary eutectic mixtures of a quaternary ammonium salt (e.g., choline chloride) acting as hydrogen bond acceptor (HBA) and a hydrogen bond donor (HBD, e.g., urea, glycerol) that have a lower melting point than their individual components. At a closer look, this definition is not sufficient and, in order to characterize eutectic systems as *deep*, a negative deviation of the melting curve from an ideal solution must be observed (see Figure 1) [3,4]. This non-ideality signifies strong attractive interactions between the DES components, which leads to lower melting temperatures as an ideal solution of both components would suggest. Moreover, the sole presence of hydrogen bonds within a mixture (e.g., as in a mixture of water and ethanol or in mixtures of carboxylic acids) is not enough to account for such a melting point depression rendering the definition of DESs being solely formed by hydrogen bonding incomplete [3]. This is further underlined by combinations of various salts and water showing significant melting point depression. This deepness of the eutectic systems comes with a few advantages as it widens the availability of substances for their usage as solvents in various applications (e.g., as extraction medium [5] or reaction environment [6]). Moreover, the strong interactions between the DES molecules can lower the reactivity of the DES components in (bio)chemical reactions [7]. The particular strong interactions within a DES can prevent a component form distorting the enzyme structure [8] and if the product forms a DES with the solvent mixture, its back reaction may be less thermodynamically favored leading to better product yields [7]. However, whether the eutectic mixture of interest as reaction medium for biocatalysis must inherit this *deepness* or whether a (non-deep/ideal) eutectic mixture also does the job, depends on the enzyme and reaction system of choice.

A popular class of DESs is based on choline chloride, which have been referred to with special names in the literature (e.g., “reline” for a mixture of choline chloride and urea, or “glyceline” for choline chloride and glycerol). This, however, can lead to the false impression that the formed eutectic mixture is a new chemical substance rather than just a mixture of two components at a certain ratio (e.g., 1:2). To circumvent this misconception, all DESs reported in this work are referred to with their individual components plus their molar ratio. For example, ChCl-Urea (1:2) is used for a mixture of choline chloride and urea with a molar ratio of 1:2 or ChCl-Gly (1:9) for a mixture of choline chloride and glycerol with a molar ratio of 1:9.

Moreover, the combination of choline chloride and glycerol or ethylene glycol with a molar ratio of 1:2 belong to the most used eutectic systems not only but also for their application in biotechnology. However, using the above-mentioned definition of a DES from the thermodynamics of the liquid mixture (negative deviation from ideality) these mixtures have been debated to not fulfill this condition [3]. Mixtures of choline chloride and fatty alcohols rather result in a positive deviation from ideality or near ideal behavior suggesting they should not be called a *deep* eutectic solvent [3,9]. While definite proof for choline chloride combined with glycerol or ethylene glycol is still missing, their mixing properties rather hint at a positive deviation [10] possibly excluding them from the DESs [3]. On the other hand, these mixtures have been widely framed as DESs in various publications. Due to preserving recognizability and their wide usage we will include them in this review, although labeling them as *deep* eutectics should be taken with care.

### 1.2. Enzymes and Proteins in Deep Eutectic Solvents

Although proteins and enzymes typically exist in an aqueous environment probably combined with a buffer system, there has been growing interest in the use of non-aqueous solutions for their storage, separation or as reaction medium. This expands the range of systems available for their application, but also necessitates a comprehensive understanding of the interactions between proteins/enzymes and different solvents. The solvent can affect the protein/enzyme in various ways [11,12,13,14]. Depending on the polarity of the solvent, proteins are less flexible in organic media [15,16] than in aqueous solution. Organic solvents may accumulate on the surface of the biomolecule stabilizing its three-dimensional structure or inducing denaturation mechanisms, which makes the study of their solvation layer important [17]. Depending on the specific protein or enzyme, at least a minimum number of structural water molecules must be present on the protein surface to maintain its structure and function in an organic solvent. The hydration of the protein is crucially linked to the thermodynamic water activity in the bulk phase [18], enabling its control. All the interactions between biomolecules, solvent, water, and reactants in multicomponent systems must be studied in detail at a molecular level. In this regard, molecular dynamics (MD) simulations are a powerful tool for studying thermodynamic and transport processes at the molecular scale. They can provide a foundation for understanding the underlying phenomena and shed light on molecular processes that are not (easily) accessible by experimental measurements. This can be of particular interest for biological systems such as proteins as it helps to understand their (un)folding behavior in different environments [11] and their interactions with smaller molecules for the optimization of for example, drug design [19] or protein stability [13,20]. Steady developments in computational hardware and modeling tools over the last decades have enabled the investigation of more biomolecular systems by means of computational analysis.

**Figure 2:**
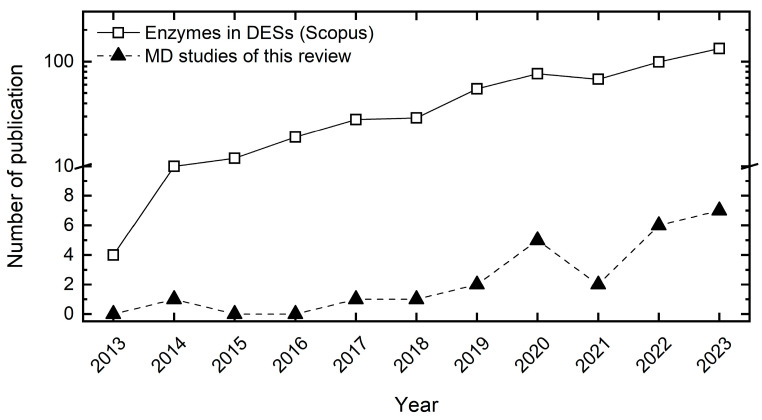
Number of publications for molecular dynamics (MD) simulations of biomolecules in deep eutectic solvents (DESs) from 2013 to 2023 (black triangles), which are reviewed within this work. Scopus [34] search quest for enzyme in deep eutectic solvents (white squares, accessed for “enzyme deep eutectic solvent” on 22 January 2024). If this search were extended to other biomolecules (DNA, cofactor), the number of publications would probably even be larger. The range from 10 onwards is displayed in a logarithmic scale for better visualization. For a summary of experimental studies dealing with enzymes in DESs, we refer interested readers to the very recent review of Zhang et al. [33].

While the assessment of organic solvents on protein/enzyme structures via all-atom MD simulations dates back to the 1990s [21,22] and has yet expanded during the past decades [15,18,23,24,25,26], the investigation of the impact of DESs on enzymes has only started less than ten years ago [8] with rising popularity during the past years. This is probably enabled by the force field adaptations and refinements of existing biomolecular models for mainly choline-based DESs starting in the early 2010s [27,28,29,30,31,32]. Figure 2 illustrates the number of publications studying biomolecules in DESs from 2013 to 2023 (black triangles). The total number of publications studying enzymes in DESs (white squares) surpasses the MD studies by more than one order of magnitude, signifying that only a small fraction of studies dealing with biomolecules in DESs apply MD simulations.

This work serves therefore as a review of recent examples in modeling and investigating the impact of different DESs on various biomolecular systems by means of all-atom MD simulations. It is to this end that this study is not restricted solely to catalytic active systems but also covers non-enzymatic proteins, DNA, and biological necessary co-factors. Furthermore, we discuss the advantages and challenges associated with MD simulations and provide guidance on what to explore and focus on in future studies of DESs for biological relevant systems. Zhang et al. [33] recently reviewed experimental studies of enzyme catalysis in different DESs.

## 2. Current State—MD Simulations of Biomolecules in Deep Eutectic Solvents 

**Figure 3:**
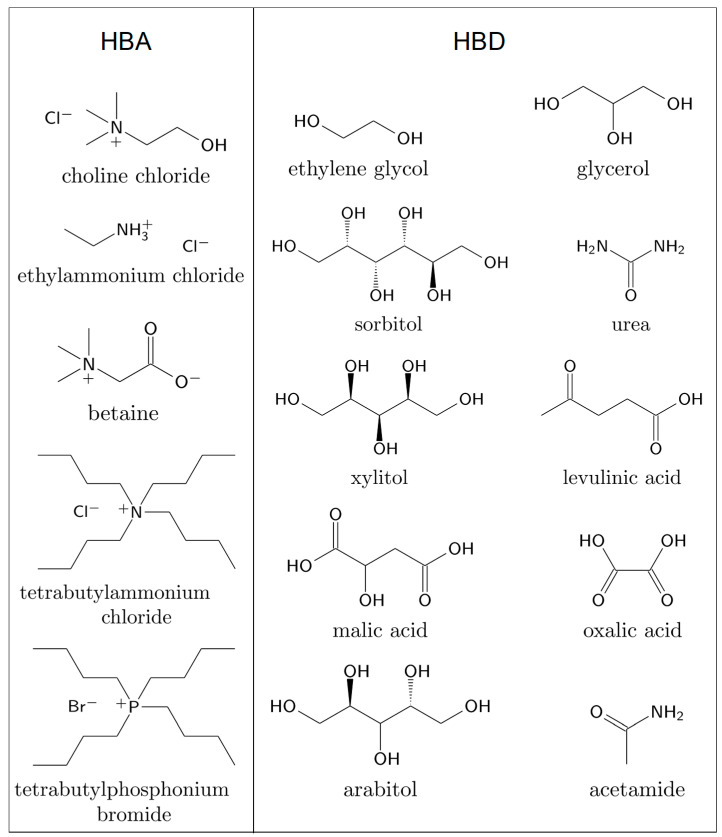
Chemical structures of hydrogen bond acceptors (HBAs) (**left**) and hydrogen bond donors (HBDs) (**right**) that form the deep eutectic mixtures reviewed within this work.

Table 1 summarizes the biomolecular systems in DESs studied with MD simulations on the atomistic scale with information about the force field that is used to model the DES components as well as the properties studied within the simulations. We may only briefly discuss articles published very recently but have included the relevant data in the figures and tables. As many DESs reported within this work are based on the quaternary ammonium salt choline chloride acting as HBA, the commonly used practice of labeling the DES components as HBA and HBD is used. In Figure 3, the chemical structures of the HBAs and HBDs of the deep eutectic mixtures reviewed within this work are illustrated. 

### 2.1. Selection of Force Fields for DESs

Before we dive into the modeling of biomolecules in different deep eutectic mixtures, we want to shortly emphasize the challenges arising from modeling the DES molecules in all-atom MD simulations. The force field accuracy is probably the most crucial step for modeling biomolecules in DESs. In other words, the simulation results are only as reliable as the underlying model. All MD simulations of biomolecules reported within this work are based on one of the four widely used biomolecular force field families namely Amber, CHARMM, GROMOS and OPLS-AA (Table 1), which have already been shown to give a similar performance [35,36,37]. Force field developments of the past decade have made MD simulations of DESs available for applications to biomolecular systems [38]; however, this is, to the best of our knowledge, limited to non-polarizable force fields. Their developments are mainly based on model refinements for ionic liquids [39]. A scaling of the point charges of ionic components was found to be necessary due to charge transfer between the components within a DES [40,41]. This observation is not unique to a specific force field family and, thus, a more universal problem of modeling ionic components with non-polarizable force fields [38]. Nevertheless, no unified charge scaling approach could be found as the scaling has in some cases only been applied to the ionic components [27,28], while others are also scaling the charges of the non-ionic HBD [29]. This basically adds another tunable parameter to the force field development for ionic systems, which has even been questioned to be necessary at all [42]. In other cases, an increase of the van der Waals diameter for the Lennard–Jones (LJ) interactions was found to be necessary in addition to a charge scaling of the ionic components to sufficiently represent the density and dynamic viscosity [43]. In general, non-polarizable force field models can reproduce many static properties such as density, heat capacity, thermal expansion coefficient, surface tension, and solvation free energy Δ*G*_solv_ with good accuracy [38,44]. However, determining dynamic properties (e.g., dynamic viscosity and self-diffusion coefficient) remains challenging, although their determination benefitted the most from the charge scaling. For a better representation of (ionic) DES molecules in MD simulations, polarizable force fields could be a promising strategy that has become more popular with increasing computational capabilities. For more information about recent force field developments and modeling of thermophysical properties of DES by different modeling approaches (incl. MD simulations), the reader is referred to other reviews [38,44].

**Table 1:** Summary of biomolecular systems in deep eutectic solvents (DESs) investigated by molecular dynamics (MD) simulations. The DES is classified by its hydrogen bond acceptor (HBA) and hydrogen bond donor (HBD) with the respective composition in the eutectic system (e.g., 1:2). The used force field for the DES molecules in the simulations is explicitly given, whereas the force fields used for the biomolecule or water, if present, are not stated. The sampling time of each simulation is added, and possible replica simulations are noted. The properties sampled in the MD simulations, that are used for understanding the behavior of the biomolecule in different solvent environments, are summarized. Abbreviations: RMSD: root mean square deviations, RMSF: root mean square fluctuations, *R_G_*: radius of gyration, RDF: radial distribution function, SASA: solvent accessible surface area.

Biomolecule Type	Enzyme/Protein/DNA	HBA	HBD	Composition HBA:HBD	Force Field for DESs	Sampling Time/ns	Properties Studied with MD Simulations	Reference
Lipase (EC 3.1.1.3)	*Candida antarctica* Lipase B (CALB)	Choline Chloride	Urea	1:2	GROMOS 43a1 [45]	50	RMSD, RMSF, *R_G_*, RDF, hydrogen bonds, protein contact maps, secondary structure	[8]
		Choline Chloride	Glycerol	1:2	GROMOS 54a7 [46]	30	RMSD, RMSF, *R_G_*, RDF, SASA, secondary structure	[47]
		Choline Chloride	Glycerol	1:2	OPLS-AA [48]	30	RMSD, RMSF, *R_G_*, RDF, hydrogen bonds, active site structure	[49]
		Betaine	Xylitol	1:1				
		Choline Chloride	Urea	1:2	OPLS-DES [29]	500	RMSD, RMSF, SASA, hydrogen bonds, secondary structure, solvation of DESs	[50]
		Choline Chloride	Glycerol	1:2				
		Choline Chloride	Urea	1:2	OPLS-DES [29]	2 × 200	RMSD, RMSF, *R_G_*, hydrogen bonds, secondary structure, active site structure	[51]
		Choline Chloride	Glycerol	1:2, 1:3, 1:4				
		Choline Chloride	Ethylene Glycol	1:2, 1:3, 1:4				
		Choline Chloride	Sorbitol	1:1	CGenFF [52,53]	100	RMSD, active center geometry and distances to ligand	[54]
		Choline Chloride	Xylitol	1:1				
		Choline Chloride	Arabitol	1:1				
Lipase (EC 3.1.1.3)	Thermoalkalophilic Lipase	Choline Chloride	Urea	1:2	CGenFF [52,53]	2 × 300	RMSF, *R_G_*, RDF, SASA, active site structure	[55]
	*Aspergillus oryzae* CJLU-3 (AOCL@CaP)	Choline Chloride	Glycerol	1:2	GROMOS 54a7 [46]	100	RMSD, RMSF, hydrogen bonds, secondary structure, active site structure	[56]
	*Bacillus subtilis* Lipase A	Choline Chloride	Acetamide	1:2	GROMOS 54a7 [46]	100	RMSD, *R_G_*, SASA, hydrogen bonds, secondary structure, spatial distribution of solvents, hydration	[57]
		Choline Chloride	Ethylene Glycol	1:2				
		Tetrabutylphosphonium Bromide	Ethylene Glycol	1:1				
Lysozyme (EC 3.2.1.17)	Hen Egg White Lysozyme (HEWL)	Choline Chloride	Urea	1:2	CGenFF [52,53]	350	RMSD, RMSF, *R_G_*, SASA, hydrogen bonds, secondary structure	[58]
		Choline Chloride	Glycerol	1:2	GAFF-based [42,59]	200	RMSD, RMSF, *R_G_*, RDF, hydrogen bonds, protein contact maps, solvation of DESs	[60]
		Choline Chloride	Urea	1:2	GROMOS 54a7 [46]	50	RMSF, *R_G_*, RDF, SASA, secondary structure	[61]
		Choline Chloride	Urea	1:2	OPLS-DES [29]	120	RMSD, RMSF, *R_G_*, SASA, hydrogen bonds	[62]
		Choline Chloride	Glycerol	1:2				
		Choline Chloride	Ethylene Glycol	1:2				
		Choline Chloride	Levulinic Acid	1:2				
		Choline Chloride	Malic Acid	1:1				
		Choline Chloride	Oxalic Acid	1:1				
Protease (EC 3.4.23.16)	HIV-1 Retropepsin	Choline Chloride	Urea	1:2	GROMOS 43a1 [45]	500	RMSD, RMSF, hydrogen bonds, solvation of DESs	[63]
		Choline Chloride	Glycerol	1:2				
		Choline Chloride	Ethylene Glycol	1:2				
Alcohol Dehydrogenase (EC 1.1.1.1)	Horse-Liver Alcohol Dehydrogenase	Choline Chloride	Glycerol	1:2	OPLS-DES [29]	100	RMSF, RMSD, hydrogen bonds, hydration	[64]
		Choline Chloride	Glycerol	1:2	CGenFF [52,53], GAFF-DES [27,28], OPLS-DES [29]	2 × 100	RMSD, RMSF, *R_G_*, hydrogen bonds, hydration, solvation of DESs	[35]
		Choline Chloride	Ethylene Glycol	1:2	CGenFF [52,53], GAFF-DES [27,28], OPLS-DES [29]			
		Choline Chloride	Glycerol	1:2, 1:9	GAFF-DES [27,28]	2 × 100	RMSF, hydration, solvation of DESs, spatial distribution of solvents	[65]
		Choline Chloride	Ethylene Glycol	1:2	GAFF-DES [27,28]			
		Ethyl Ammonium Chloride	Glycerol	1:2	OPLS-AA [48]			
Peroxidase (EC 1.11.1.16)	Versatile Peroxidase (VP)	Choline Chloride	Glycerol	1:2	GROMOS 54a7 [46]	100	RMSD, RMSF, *R_G_*, hydrogen bonds, active site structure	[66]
Cofactor	Nicotinamide Adenine Dinucleotide (NADH)	Choline Chloride	Urea	1:2	GAFF-based [59]	300	NADH conformation and contact distances	[67]
		Choline Chloride	Glycerol	1:2				
g-DNA	Thrombin Binding G-quadruplex Aptamer (TBA)	Choline Chloride	Urea	1:2	GAFF-DES [27]	2000	RMSD, RMSF, RDF, hydrogen bonds, contact distances, solvation of DESs, torsion angles, spatial distribution of solvents	[68]
g-DNA	C-Kit Oncogene Protomer G-quadruplex DNA	Choline Chloride	Urea	1:2	GAFF-DES [27]	2000	RMSD, RMSF, hydrogen bonds, spatial distribution of solvents	[69]
Other Proteins	Chicken Villin Headpiece Subdomain (HP-36)	Choline Chloride	Urea	1:2, 1:5	OPLS-AA [48]	2 × 500	RMSD, *R_G_*, SASA, hydrogen bonds, secondary structure, solvation of DESs (incl. interaction coefficients), spatial distribution function of solvents	[70]
	Bovine Serum Albumin	Choline Chloride	Urea	1:2	CGenFF [52,53]	350	RMSD, RMSF, *R_G_*, SASA, hydrogen bonds, secondary structure, protein contact maps	[71]
	Human VH Antibody Fragment HEL4	Choline Chloride	Glycerol	1:2	GAFF-based [42,59]	200	RMSD, RMSF, *R_G_*, hydrogen bonds, protein contact maps, solvation of DESs	[60]
	*β*-Lactoglobulin	Betaine	Sorbitol	2:1	GAFF-based [42,59]	400	RMSD, RDF, *R_G_*, SASA, hydrogen bonds, secondary structure, solvation of DESs, spatial distribution functions, interaction energies	[72]
	Amyloid *β*_42_ Monomer	Tetrabutylammonium Chloride	Ethylene Glycol	1:3	CGenFF [52,53]	1000	RMSD, RDF, SASA, hydrogen bonds, secondary structure, contact maps, free energy landscape	[73]
	Ubiquitin	Betaine	Glycerol	1:2	GAFF-opt [43]	5 × 500–1000, 2 × 2000	RMSD, *R_G_*, SASA, secondary structure, hydration, solvation of DESs, torsional dynamics, coordination numbers	[43]

In Table 1, the force field choices for the DES molecules in the reviewed MD studies are summarized. Applying all four well-known biomolecular force fields for modeling the DES constituents, some studies used specifically refined DES force fields (e.g., GAFF-DES [27,28], OPLS-DES [29]), while others utilized the respective small molecule force fields without any refinement towards deep eutectics (e.g., CGenFF [52,53], GROMOS 54a7 [46]). When selecting a force field for the desired simulations set-up, its performance for representing different bulk properties of the DESs in the simulations as well as its limitations should be kept in mind. In particular, the impact of these bulk properties on the sampled enzyme properties should be carefully evaluated and considered when interpreting the results of the MD simulations; this can for instance include the impact of an underestimated dynamical behavior on enzyme flexibility in a DES. The large (and by many force fields highly overestimated [35,38]) viscosity of DESs in MD simulations can also make the equilibration and sampling of those highly viscous systems challenging. This may necessitate long sampling times and special equilibration schemes. Different procedures for equilibrating the DES solutions have been proposed. For instance, a temperature annealing procedure [35,64,65] based on the equilibration of ionic liquids [74] as well as a pressure coupling/decoupling procedure [27,28,30], which originally comes from the equilibration of polymer solutions [75], have been proposed.

### 2.2. Hydrolases

For the enzyme class of hydrolases (EC 3), MD studies exist only for the subclasses of the widely studied lipases [8,47,49,50,51,54,55,56,57] (EC 3.1.1.3) acting on carboxylic ester bonds, lysozyme [58,60,61,62] (EC 3.2.1.17), an antimicrobial enzyme that is part of the innate immune system of animals, and one study of the HIV-1 protease [63] (EC 3.4.23.16).

#### 2.2.1. Lipases

Due to their significance in biotechnological applications and their robustness towards different organic solvents, lipases have been the most studied enzyme class regarding their employment in deep eutectic systems via experiments and MD simulations (see Table 1). *Candida antarctica* Lipase B (CALB) stands out as the most studied enzyme, notably being the subject of the first all-atom molecular dynamics study of an enzyme in a DES, namely ChCl-Urea (1:2), carried out by Monhemi et al. [8]. They investigated the denaturation of CALB in an aqueous 8 M urea solution and proposed that urea distorts the enzyme structure through directly replacing intra-protein hydrogen bonds with protein–urea hydrogen bonds. This leads to clear evidence of protein unfolding as seen in the increasing root-mean-square-deviations (RMSD) and radius of gyration (*R_G_*) in the MD simulations, even after a rather short sampling time of 50 ns. In comparison, the secondary structure and active site architecture of CALB could be stabilized in ChCl-Urea (1:2), despite its high urea content. This explains the experimental results of Gorke et al. [7], who observed a deactivation of CALB in aqueous urea solution but a stabilization of CALB in ChCl-Urea (1:2). The strong interaction of urea with the other DES components (choline and chloride), through hydrogen bonds or salt bridges, prevents urea from denaturing CALB. This exemplifies the potential impact of deep eutectics having a negative deviation from thermodynamic ideality, which results from highly attractive interactions between the DES constituents, on enzymes. This effect is further enhanced by hydrogen bonds between urea, choline, and chloride with CALB’s surface residues stabilizing the enzyme structure in ChCl-Urea (1:2). With their findings, the impact of (deep) eutectic mixtures on biomolecules resulting from synergy and complex formation were established [8].

Nian and coworkers [47,49] studied CALB in the presence of ChCl-Gly (1:2) (with additives) and Betaine-Xylitol (Bet-Xyl) (1:1), revealing their impact on different reactions. They found a synergistic effect exerted by CALB and ChCl-Gly (1:2) for the enzymatic synthesis of lauroyl glycine. This is significantly diminished when CALB is used in classical organic solvents and when the catalysis is taking place without the enzyme in different DESs [47]. The activity of CALB could be further improved by the addition of MgCl_2_ hydrate; a component also widely used for the formation of DESs. The secondary structure of CALB was found to be conserved in the MgCl_2_-ChCl-Gly mixture and the interactions of chloride with the acyl binding pocket of CALB highlights its importance for improved substrate binding. They suggest a less important role of the CALB structure in its improved reactivity and related the interactions of the DES medium with lauric acid to an increased electron affinity [47]. The same authors further investigated CALB with the help of molecular simulations in ChCl-Gly (1:2) and Bet-Xyl (1:1) and ethanol as control medium [49]. The catalytic performance of CALB—especially its thermal stability—could be significantly improved in the presence of glycerol-containing DESs compared to the aqueous control. A lower conformational flexibility of CALB, quantified with root-mean-square-fluctuations (RMSF), in the DES solutions hints towards its improved stability, while its global structure remained folded in the simulations. Neither CALB’s active triad nor its acyl binding pocket was significantly changed by the presence of ChCl-Gly (1:2); however, interactions of chloride and choline with the acyl pocket were noted. This suggests that CALB is not activated by increasing the size of its active pockets, but by synergistic solvation effects of DES components and substrate [49]. In parallel with their previous work [47], strong hydrogen bonding between the substrate and DESs results in improved electron-attracting capacity which was not present in the pure glycerol control. However, the sampling times of 30 ns in both studies are the shortest amongst all reported MD studies, which may be enough for the solvent molecules to properly solvate the enzyme structure (and its active site) but could lack evidence of its conformational changes in a highly viscous mixture. Nevertheless, the usage of two different force fields (GROMOS54a7 [46] and OPLS-AA [48]) between both publications resulting in similar simulation outcomes adds supporting proof to their results.

Using a refined force field for DESs (OPLS-DES [29]) in simulating CALB in DESs, Qiao et al. [50] discovered a preferential interaction between CALB and HBD (urea and glycerol) in DESs compared to choline chloride. This led to subsequent accumulation of the respective HBD on the enzyme surface, which was also observed in other MD studies [20,76]. This accumulation induced a (de)stabilization and suggested a more significant role of the HBD in the enzyme function than choline [50]. Furthermore, the addition of water filled the gaps between the larger DES molecules on CALB’s surface instead of replacing them. The presence of water weakened the hydrogen bonding interactions between the enzyme and DESs in the first solvation shell, which probably promoted ligand interactions with the enzyme. Examining CALB’s active triad (Ser105, Asp187 and His224) revealed varying outcomes of ChCl-Gly (1:2) and ChCl-Urea (1:2) and their mixtures with water. Adding 25 mol% water in ChCl-Gly (1:2) reconfigured the secondary structure of the active triad to align with its conformation in aqueous solution. In contrast, this effect was less evident in ChCl-Urea (1:2). In total, the presence of DES molecules in the solvation shell did not markedly alter the rest of CALB’s secondary structure [50], which is based on longer simulations times (500 ns sampling) compared to prior MD studies of CALB.

Kovács et al. [51] employed MD simulations of CALB not only in choline-chloride-based DESs (HBDs: urea, glycerol, and ethylene glycol (EG)) with a molar ratio of 1:2 but also investigated ChCl-EG and ChCl-Gly at different molar ratios of 1:3 and 1:4. They found CALB’s structure to be stable within the MD simulations (RMSD and *R_G_*) for all DESs, except for ChCl-Urea (1:2). Although the RMSD is lower in ChCl-Urea (1:2) compared to aqueous solutions with and without 8M urea, it did not reach a constant value within 200 ns contrary to the simulations of Monhemi et al. [8], Nian et al. [47,49], (using shorter sampling times) and more remarkably Qiao et al. [50], albeit using the same force field as the latter one. Moreover, the largest discrepancies between the MD studies occurred for the aqueous urea solution. They attributed the differences with the various force fields used and suggest that some force fields require more time for equilibration of the protein structure. In addition, a shift of the HBD ratio in the DESs did not change the global enzyme structure with slightly larger RMSD values observed as for the 1:2 molar ratio. RMSF profiles revealed the flexibility of the lid that controls the active site access as highest amongst all residues. The flexibility of this region is dampened in the DES systems, which is consistent with the results from Monhemi et al. [8] and Nian et al. [49]. The intra-protein hydrogen bonds are preserved in all tested DES solutions compared to alterations in the hydrogen bonding network within CALB appearing in water, n-hexane, and urea. The secondary structure of CALB and the lid as entrance to the active site are impacted by the DESs in a similar way to the aqueous solution. Moreover, the DES molecules did not interfere with the three-dimensional structure of the active site triad. Hence, Kovács et al. [51] concluded that the changes in reaction rate are due to solvent–substrate interactions and are not caused by solvent–enzyme interactions.

Regarding lipases other than CALB, only a few notable MD studies exist for e.g., a thermoalkalophilic lipase [55] and a lipase nanohybrid from *Aspergillus oryzae* [56]. Shehata et al. [55] investigated two equilibrium conformations (open and closed lid) of a thermoalkalophilic lipase in ChCl-Urea (1:2) at varying hydration levels (30 and 70 mol%) and different temperatures (310 K and 373 K). ChCl-Urea solutions could stabilize the enzyme structure—in particular, three regions: n-terminal, lid domain and b-flap—indicated by a decreased RMSF under elevated temperatures, while preserving the global lipase structure. This effect is reduced for hydrated DESs; however, a thermostabilizing effect is still expected. In addition, the three-dimensional structure of the catalytic active triad of the thermoalkalophilic lipase (Ser114, Asp318 and His357) remains intact in the DES solutions. A combination of ChCl-Urea (1:2) and water even improved the catalytic activity and thermal stability of the lipase, which highlights the importance of testing different water levels to optimize the solvent impact in biocatalytic reactions. Based on their MD simulations, the presence of water on the enzyme surface is therefore necessary to activate the lid and b-flap domains, which could then stimulate the enzyme function [55].

Zhang et al. [56] investigated the formation of a nanohybrid of lipase from *Aspergillus oryzae* and calcium phosphate at the interface of a ChCl-Gly (1:2)/water system. It must be noted that the two-phase system of the mixture containing water, choline chloride and glycerol observed in their work should result from the slow mixing behavior of these components due to the large viscosity of ChCl-Gly (1:2). As water is completely miscible in ChCl-Gly (1:2) solutions [65], it is therefore presumably not a thermodynamically stable solution. With the help of MD simulations, they could relate the presence of Ca^2+^ ions and a combination of Ca^2+^ and ChCl-Gly (1:2) to a shifted secondary structure of their lipase from an alpha-helix-rich to a beta-sheet-rich structure. This impacted the conformation of the active site residues (Ser146, Asp201 and His258) and overall stabilized the enzyme structure in these solutions [56].

Collectively, these studies of lipases in different DESs underscore the potential of DESs in augmenting enzyme stability and activity for lipase-catalyzed reaction, which has recently been extended to different mutations of lipase A from *Bacillus subtilis* [57] and to polyol-based DES as substrates for CALB [54]. DESs exert a synergistic effect on lipases, which could be alleviated by additional substances [47], thereby, stabilizing its active triad and lid domain in these mixtures. This led to improved catalytic activity and thermal stability in most of the tested systems. However, it is suggested that this improvement in enzyme activity is rather due to solvent–substrate than to solvent–enzyme interactions [47,49,51], which could also be found in other cases [77].

#### 2.2.2. Lysozyme

Kumari et al. [58] presented the first investigation of lysozyme from hen egg white in a DES—namely ChCl-Urea (1:2)—and its mixtures with water. Its structure is partially folded but slightly swollen (increased *R_G_*) in all ChCl-Urea (1:2) mixtures with water; however, it exhibits a larger deviation from its crystal structure compared to an aqueous solution. Thereby, a mixture with a large water content of 50 mol% water showed the least stability (largest RMSD) in the simulations. Furthermore, the presence of DES molecules on the enzyme surface increased the solvent accessible surface area (SASA) by notably shifting its hydrophobicity. This also affected the secondary structure of lysozyme by mainly resolving the *β*-sheets and half of the *α*-helices. These changes are probably induced by a preferred interaction of lysozyme with the choline cation via hydrogen bonds in pure ChCl-Urea (1:2), while the hydrogen bonding with urea dominates in the DES water mixtures. Notably, the flexibility of lysozyme (in terms of RMSF) was drastically reduced in pure ChCl-Urea (1:2) compared to the aqueous solution; however, it significantly exceeded the values of the aqueous case in ChCl-Urea (1:2)/water mixtures with different regions of the enzyme becoming more flexible. This suggests that the combination of DES and water can destabilize the enzyme, while it seems stabilized in a pure DES environment [58].

Parisse et al. [60] investigated lysozyme and the human VH antibody domain (subject to a later chapter) in a mixture of choline chloride and glycerol (molar ratio 1:2) at 20 wt.% water. Before analyzing the proteins, they compared different force fields for the DES including the commonly applied charge scaling method with a self-derived scaling factor of 0.92, a refining of the LJ parameters from Chaumont et al. [42], and a novel approach using different LJ parameter combinations depending on the interaction (DES-DES from refined LJ parameters [42] vs. DES–water–protein from original GAFF [59]). Based on pair distribution function for pure ChCl-Gly (1:2), they found a better agreement for the force field with reparametrized LJ parameters compared to the scaled model, confirming the approach of Chaumont et al. [42]. The hydrated DES mixtures preserved the original crystal structure of lysozyme in all three tested force fields showed by a reduced RMSD compared to the aqueous solution, while its secondary structure remained intact. In line with other studies of enzymes in ChCl-Gly (1:2), lysozyme’s conformational flexibility is reduced in the DES/water mixture in three distinct regions. However, a dynamical cross-correlation matrix suggests that lysozyme’s function may not be affected by its rigidity. A preferred interaction of the choline cation with two tryptophan residues—in particular for the mixed force field—could be confirmed, which were present in a measured crystal structure in ChCl-Urea (1:2), highlighting its importance in the crystallization of lysozyme in choline-based DESs [78]. Contrary to Kumari et al. [58], the hydrogen bonding with water is not weakened by the presence of high concentrations of DES, which contributes to further stabilization of the protein.

Hebbar et al. [62] studied lysozyme in six different choline–chloride-based DESs (with the HBDs: urea, glycerol, ethylene glycol, levulinic acid (LevAc), malic acid (MalAc), and oxalic acid (OxAc)), adding a huge variety of systems to study its interactions in different media. With the exception of ChCl-Gly (1:2) and ChCl-OxAc (1:1), lysozyme exhibits a much lower RMSD value in DESs compared to the simulation in pure water. This finding contradicts the results of Kumari et al. [58], who observed an opposite trend using CGenFF [52,53] to model the DES molecules in the MD simulations as opposed to OPLS-DES [29] in the study of Hebbar et al. [62]. This difference is also visible in the radius of gyration (~1.40 nm) for ChCl-Urea (1:2), which is much lower compared to Kumari et al. [58] (~1.65 nm) and the experimental value (1.78 ± nm) [79]. However, the *R_G_* of lysozyme in different DES was quite close to its value in water (around 1.4 nm) except for ChCl-LevAc (1:2) [62]. The flexibility of the residues in proximity of the active site (residues 35–52) have been drastically reduced in all DESs except for ChCl-OxAc (1:1). With the exception of a few specific residues, the flexibility of lysozyme is reduced in DES solutions compared to water. This is in line with Parisse et al. [60], who used a GAFF-based force field for modeling the DES, although different water contents of the DES were employed. Furthermore, an end-to-end distance analysis revealed that lysozyme in ChCl-OxAc (1:1) and ChCl-MalAc (1:1) exhibits distortions of its secondary structure. This is further confirmed by an increase in its surface area in both DESs. In contrast to Kumari et al. [58], the HBD dominated the hydrogen bonding interactions with lysozyme in all tested DESs with slight variations in line with the hydrogen bonding capacity of the respective HBD. 

The study of lysozyme in DESs has recently been expanded by Kaumbekova and Shah [61]. In conclusion, many of the tested pure DESs mixture can stabilize the lysozyme structure. However, the interpretation of aqueous mixtures with DESs diverge as different structural values (RMSD and *R_G_*) for different force fields have been observed in the studies. While Kumari et al. [58] suggest a destabilizing effect of ChCl-Urea (1:2)/water mixtures on lysozyme, which is in agreement with their study of bovine serum albumin [71], this effect is not visible in other studies [58,59,60]. 

#### 2.2.3. Protease

Recently, Padariya et al. [63] studied HIV-1 protease, an important enzyme in the life cycle of the HIV virus, in ChCl-Urea (1:2), ChCl-Gly (1:2) and ChCl-EG (1:2) revealing greater stability of the protease in ChCl-Urea (1:2) compared to the other DESs. The introduction of darunavir ligand induced protein–protein interactions in particular for the urea-based DES improving ligand binding. Using the GROMOS 43a1 [45] force field for the DES molecules, they observed a phase separation for aqueous mixtures of ChCl-EG (1:2) and ChCl-Gly (1:2) that is not present in the experiments and may as such be an artifact of the force field similarly to the observed phase separation for ChCl-Gly (1:2)/water mixtures using OPLS-DES [35].

### 2.3. Oxidoreductases

Oxidoreductases (EC 1) are a widely used enzyme class for catalyzing redox reactions by inducing the electron transfer from one molecule to another. For this enzyme class, MD studies in deep eutectic mixtures of alcohol dehydrogenase [35,64,65] (EC 1.1.1.1) and versatile peroxidase [66] (EC 1.11.1.16) exist. Despite not being an oxidoreductase itself, a study of nicotinamide adenine dinucleotide [67] (NADH) cofactor—a vital part for electron transfer in many redox reactions catalyzed by oxidoreductases—in DESs is added in this chapter.

#### 2.3.1. Alcohol Dehydrogenase

Our group [35,64,65] has investigated different DESs used as a reaction medium for the redox catalysis carried out by alcohol dehydrogenase (ADH) from horse liver. This includes an investigation of horse liver ADH in the DES ChCl-Gly (1:2) and its mixtures with water using both experimental measurements and molecular modeling techniques [64]. Compared to some other studies, here a direct comparison of MD and experimental results are made. The MD simulations revealed that ADH experienced significantly reduced hydration and conformational flexibility in highly concentrated ChCl-Gly (1:2) solutions, which both correlated with the reduced thermodynamic activity of water *a*_w_ in these mixtures. The hydration of ADH was defined by a favorable interaction between water and the DES molecules, as water remains in the bulk phase rather than populating the enzyme surface. The concept of enzyme hydration, dependent on *a*_w_, has already been explored for enzymes in organic solvents [18]. The presence of water on the surface and at the active center of ADH is crucial for the reaction mechanism explaining the poor reactivity of ADH in highly concentrated ChCl-Gly (1:2) mixtures. The RMSD of ADH in the DES solutions was significantly lower than its value in an aqueous solution indicating a considerable influence of the DES environment on ADH’s structure. Otherwise, the intra-protein hydrogen bonds suggest that the structure remains intact, and the enzyme deactivation is not caused by a denaturation of its structure.

Motivated by these results and discussions regarding force field developments for the emerging solvent class of deep eutectics, we conducted a comparison of different force fields (CGenFF [52,53], GAFF-DES [27,28], and OPLS-DES [29]), which have already been applied for MD simulations of DESs [35]. The efficacy of each model was tested for DES/water mixtures considering their bulk phase properties and interactions with ADH. While significant differences in model performance were observed for the dynamic viscosity particularly for the pure DESs (significantly improved by the charge scaling within GAFF-DES [27,28] and OPLS-DES [29]), all models provided similar accuracy regarding static bulk properties. Additionally, the tested force fields gave similar results for the studied protein properties (RMSF, RMSD and intra-protein hydrogen bonds) as well as protein-solvent/protein-water interactions. ADH exhibited a significant decrease in RMSD and RMSF in the DES solutions suggesting a rigid structure that is closer to its crystal structure as in aqueous solution. Notably, the substantial difference in the viscosity of the DES solutions among the tested force fields did not affect the flexibility of ADH in the simulations. The greatest discrepancies between the force fields were found for the aqueous solution, OPLS-AA/M [48,80] displaying a considerable lower RMSD compared to CHARMM36m [81] and Amber03* [82]. This difference could be attributed to the protein force fields. The *R_G_* decreases in DES mixtures and increases when the water content rises except for OPLS-DES. In this case, the *R_G_* remains nearly constant and has a significantly lower value in the aqueous environment compared to CHARMM36m [81] and Amber03* [82]. The solvation layers exhibit also a discrepancy between the tested force fields. Although all solvation properties show a similar trend with changing water concentration, there are quantitative differences in solvent contacts with chloride and glycerol/ethylene glycol. In the case of chloride, these differences could be attributed to different LJ parameters in the used force fields. The exception is OPLS-DES [29] for ChCl-Gly (1:2) mixtures with water, as it showed a non-physical two-phase system at increasing water concentrations. Nevertheless, the similar results for ADH hydration, RMSD, and RMSF for GAFF and CHARMM validated our earlier findings [35,64].

We then expanded our previous research by a larger variety of DES-water mixtures in a wide concentration range to experimental and computational analysis [65]. We, thereby, also focused on evaluating the specific effect of the DES components on the catalytic performance of ADH. The results were consistent with Huang et al. [64], as the reduced hydration of HLADH and, hence, its reduced flexibility was observed in all tested DES (ChCl-Gly (1:2), ChCl-EG (1:2), and EACl-Gly (2:3)). The catalytic performance of ADH in these solutions may be hindered due to the attractive interaction of the bulk phase with water molecules that effectively strips water from the enzyme surface. Spatial distribution functions indicate a preferable interaction of the enzyme surface with glycerol, which could explain the improved stability of ADH in ChCl-Gly (1:2) at 72 mol% water compared to 100 mol% water. While water continuously replaced ChCl on the enzyme surface, the interaction with the glycerol (predominantly through hydrogen bonding) remains preserved with increasing water concentrations. This leads to a stabilizing effect in glycerol containing DESs. Although not as pronounced as in ChCl-Gly (1:2), a similar trend was observed in EACl-Gly (2:3). This effect could even be alleviated by shifting the choline chloride–glycerol ratio to 1:9 [65], which demonstrates the importance of testing different HBA:HBD ratios to optimize the DES impact on biocatalysis.

#### 2.3.2. Versatile Peroxidase

Mamashli et al. [66] studied versatile peroxidase (VP) in pure ChCl-Gly (1:2) using MD simulations. They found only minor conformational changes in the enzyme structure, which contrasts with experimental measurements of hydrated DES solutions by circular dichroism, UV–vis, and fluorescence spectroscopy. These experiments suggest exposure of hydrophobic sites on VP, possibly leading to agglomeration of the protein as well as significant changes in VP’s secondary structure. However, the microstructure and hydrogen bonding network of the heme region, which is of utmost importance for the enzymatic activity of VP, were affected by the DESs in the MD simulations. This is also present in the experimental observations [66]. Lower concentrations of ChCl-Gly (1:2) might be beneficial for VP catalysis, as they can introduce some minor conformational changes of the enzyme structure—especially near the heme region—and even stabilize the intermediate complex formed during the reaction. 

The RMSD and RMSF of VP in ChCl-Gly (1:2) from Mamashli et al. [66] were found to be almost identical to their value in aqueous solution, with only the radius of gyration being slightly lower. On the other hand, most of the other MD studies indicate large changes in the RMSD and a significant reduction of the RMSF of parts of enzyme structures for highly concentrated DES solutions, regardless of the enzyme studied. Unfortunately, a detailed MD study in hydrated DES is lacking to evaluate the simulation results in pure DES, to compare them to the experimental measurements and to conclude whether these discrepancies in the MD simulations are due to model artifacts or to a combined effect of water and DES molecules.

#### 2.3.3. Nicotinamide Adenine Dinucleotide Cofactor

As the co-enzyme NADH is an important biomolecule for hydrogen transfer in redox reactions of oxidoreductases, it is discussed within this chapter. It is used in many biocatalytic reactions and for analytical and diagnostic purposes, although it is quite expensive. Since NADH is not stable in aqueous solution, we would like to highlight the MD study of the stability of NADH in ChCl-Gly (1:2) and ChCl-Urea (1:2) by Radović et al. [67]. Screening different betaine- and choline-chloride-based DESs revealed ChCl-Urea (1:2) as best medium for NAD(H) storage, which was further analyzed (together with ChCl-Gly (1:2), which provides a moderate improvement over water) via MD simulations and density functional theory (DFT) calculations. In aqueous solution, both forms of the cofactor (NADH and NAD^+^) quickly rearrange into a stable folded structure with the pyridine and adenine rings close to each other. This rearrangement, confirmed by DFT calculations, exposes the phosphate groups for their hydrolysis and, thus, favors degradation. In contrast, NAD(H) favors a more open conformation in the tested DES without a stacking of the aromatic rings, which in the case of ChCl-Urea (1:2) is even stabilized by intramolecular hydrogen bonds involving the vulnerable phosphates. Their solvation is significantly altered in the presence of DESs compared to the aqueous solutions, with the HBDs dominating the hydrogen bonds with the phosphate groups. In ChCl-Gly (1:2), the first solvation shell of NAD(H) consists almost exclusively of glycerol molecules. Based on DFT calculations, the experimentally observed difference between ChCl-Gly (1:2) and ChCl-Urea (1:2) in NAD(H) stability is attributed to a lower nucleophilicity of urea compared to glycerol, which reduces its ability to degrade NAD(H). In conclusion, the study of Radović et al. [67] emphasizes that for the optimization of relevant NADH-dependent dehydrogenase reactions both the solvent impact on the enzyme structure and its impact on cofactors should be considered.

Concluding the investigation of oxidoreductases and their essential cofactor NAD(H) in different mixtures, high concentrations of DESs exert a negative effect on their enzymatic activity [35,64,65,66]. In contrast, both forms of the cofactor NAD(H) seem to be stabilized in ChCl-Urea (1:2), ChCl-EG (1:2) and to some extent in ChCl-Gly (1:2) [67]. Low concentrations of ChCl-Gly (1:2) in aqueous solution have, on the other hand, shown to be beneficial for oxidoreductases improving their activity and stability. However, it is suggested that this effect occurs mainly due to the presence of glycerol [65] and the stabilization of NAD(H) [67].

### 2.4. g-DNA

Pal and Paul [68,69] added two guanine-rich DNA structures to the analysis of biomolecules in ChCl-Urea (1:2) and its mixtures with water, highlighting their importance for applications in medicine and nanotechnology. The thrombin-binding g-quadruplex aptamer (TBA) was stabilized and less rigid in highly concentrated DES solutions, while it deviated from the NMR structure in DES/water mixtures. This was due to a stabilization of its backbone torsional angles in ChCl-Urea (1:2) resulting from favorable interactions with urea. Similar to the observations of protein [60,71] and cofactor stability [67], their results suggest a beneficial use of ChCl-Urea (1:2) as a storage medium for TBA [68].

In a second study, they evaluated the thermal stability of c-kit oncogene protomer g-quadruplex DNA (c-kit DNA) in ChCl-Urea (1:2) and water [69]. While the c-kit DNA was stable up to 450 K in ChCl-Urea (1:2), it quickly unfolds in aqueous solution at the same temperature as indicated by increasing RMSD, *R_G_*, and RMSF of the DNA backbone. The c-kit DNA conformation is stabilized by hydrogen bonds with choline and urea, which decrease with increasing temperature, and by string interactions with the neutralizing K^+^-ions at elevated temperatures. These interactions induce stable *π*-*π* stacking within the DNA further preventing its unfolding. In parallel with their previous work [68], ChCl-Urea (1:2) is confirmed as a stabilizing agent to prevent temperature-induced denaturation of g-DNA [69].

In summary, we would like to highlight the thorough nature of both MD studies, as they provide many details regarding the conformations of g-DNA in DESs and are based on exceptionally long simulation times of 2 μs.

### 2.5. Non-Enzymatic Proteins

Further MD studies of different proteins in DESs include chicken villin headpiece subdomain (HP-36) [70], bovine serum albumin (BSA) [71], and the human VH antibody fragment HEL4 [60].

Sarkar et al. [70] compared the stabilizing effect of choline chloride and the ionic liquid triethylammonium acetate (TEAA) in the presence of high urea concentrations on chicken villin headpiece subdomain HP-36. While small amounts of TEAA were sufficient to counteract the denaturizing effect of urea in the solutions, the stabilizing effect of choline chloride in presence of urea reported elsewhere [8] was only present in the eutectic composition. For a lower concentration, an increase in the RMSD, *R_G_* and surface area over the rather long simulation time of 500 ns comparable to the aqueous urea solution, however, slightly dampened, could be observed. The distortion of the secondary structure and, hence, its intra-protein hydrogen bonding network of HP-36 was correlated with the urea content in the simulations, whereby the presence of TEAA countered this effect even at low concentrations while choline chloride was only able to preserve HP-36′s structure at the eutectic composition. This underlines the necessity of studying the impact of (deep) eutectic solvents not only at their eutectic mixture, but also at different HBA:HBD ratios as already demonstrated elsewhere [51,65]. The authors also calculated binding numbers of the DES molecules and their preferential interaction coefficient considering a difference in the direct protein solvation compared to the bulk phase concentrations. The backbone HP-36 preferentially interacts with urea in all tested compositions, which is drastically reduced in the presence of TEAA and eutectic ChCl-Urea (1:2), further explaining the resulting structural properties. On the other site, an exclusion of all ionic components from the protein surface could be observed. These interactions are visualized with the aid of three-dimensional spatial distribution functions resolving the main interaction sites around the protein [70].

Similar to their investigation of lysozyme [58], Kumari et al. [71] conducted MD simulations of bovine serum albumin (BSA) in ChCl-Urea (1:2) with varying hydration levels. They found a stable configuration of BSA in aqueous solution and in ChCl-Urea (1:2), while a mixture of both resulted in destabilizing effects (increased RMSD and *R_G_*) and rearrangement of the three BSA domains. Comparable to their study on lysozyme in ChCl-Urea (1:2)/water mixtures, the RMSF was the lowest in the pure DES system, while it was increased in DES/water mixtures compared to the aqueous solution. The RMSD and RMSF behavior is in agreement with their earlier study on lysozyme, which could not be reproduced by others [62]. Intra-protein contact maps revealed a change in the protein hydrogen bond network but still suggest a folded state of BSA in the DES/water mixtures. The depression of the intra-protein hydrogen bonding—and in particular the interactions between the backbone with itself, which is regulating its secondary structure—is observed in the presence of ChCl-Urea (1:2). This resulted in decreased *α*-helices in the secondary structure and a shift towards 3_10_-helices signaling a change in the overall structure of BSA while remaining in a folded state. A re-equilibration in aqueous medium after the simulations in DES mixtures suggests that a recovery of the secondary structure is possible [71].

In addition to lysozyme, Parisse et al. [60] studied the behavior of the human VH antibody fragment HEL4 in the presence of ChCl-Gly (1:2) at 20 wt.% water with three different force field parametrizations. HEL4 remains in a compact and stable conformation (evident from low RMSD and *R_G_*) in the hydrated DES closer to the crystal structure compared to the aqueous solution, while preserving its secondary structure. Like other proteins in ChCl-Gly (1:2), its structure is stabilized and its flexibility is drastically reduced dampening its dynamical behavior by 8%. The three binding sites of glycerol, which are evident from the crystal structure [78], could be confirmed with the MD simulations—in particular for the force field with mixed LJ parameters. In addition, the hydrogen bonding interactions with water are preserved in the DES/water mixture comparable with their study of lysozyme. Overall, the presence of DES showed a stabilizing effect on HEL4 improving its crystallization, this finding was observed to a lesser extent compared to other enzymes [60].

The structures of all non-enzymatic proteins in different DESs show a stabilizing effect of highly concentrated DESs solutions, which makes them attractive as stabilizing agents or for improving protein crystallization. This study of non-enzymatic proteins has recently been expanded to the amyloid *β*_42_ monomer [73], *β*-lactoglobulin [72] and ubiquitin [43] showing different effects of the DES on protein stability.

### 2.6. General Remarks

Our analysis of recent advances in modeling biological relevant macromolecules in different (deep) eutectic mixtures revealed, in part, discrepancies between different studies of the same system (enzyme + DES). These results may be explained by the assumptions and main issues associated with MD simulations, e.g., the search problem, or the force field problem [83,84,85]. All four widely used biomolecular force fields have been applied to at least some of the investigated systems (see Table 1). Some systems have even been investigated via MD simulations by using more than one force field, which cross validates the findings of the MD studies. However, differences in the protein properties have been reported for similar systems. While RMSD and RMSF are widely used to study the effect of solvents on the conformation and flexibility of proteins, their comparison with other studies using different set-ups (initial configurations from the Protein Data Bank (PDB), sampling times and times used for averaging the properties) is challenging. The calculation of an RMSD needs a reference (mostly a crystal structure from an aqueous solution e.g., from the PDB), where a change in the reference structure (e.g., the initial structure of the sampling phase, or a crystal structure in the DES as used by Parisse et al. [60]) may result in completely different behavior of the RMSD. While the RMSD remains of interest for validating the convergence of the simulations of proteins, its comparison between different studies can result in discrepancies (e.g., as seen for CALB [8,47,49,50,51]) that may not be explained solely by the use of different force fields. Moreover, the interpretation of structural properties from the MD simulations without the experimental knowledge might be challenging. For instance, low RMSD and RMSF values were associated with improved (thermo)stability for many lipases in DESs [47,49]. On the other hand, similar low flexibility and RMSD values were obtained for ADH and correlated with poor enzyme (thermo)stability [64,65]. In general, the RMSF of proteins in organic media is lower compared to aqueous solution and has been shown to depend on the polarity of the solvent [15]. However, its implications for the catalysis are different depending on the enzyme type and structure.

In addition, the conformational flexibility of proteins—expressed in RMSF—may be impacted by the sampling time and time used for averaging the RMSF making its comparison with other studies difficult. In Table 1 the sampling times of the reviewed MD simulations are summarized and vary from quite short simulations of 30 ns for a few lipase systems [47,49] to 2 μs in case of g-DNA [68,69]. Some of the studies even employed replica simulations [35,43,51,55,65,70], although their results are only used for internal validation and not shown in the publication in some cases. Due to the statistical mechanics nature of the MD simulations, it is of crucial importance to validate the results from a single dynamical simulation and perform replica simulations for the DESs systems starting from different initial configurations [86]. It has already been shown that performing shorter but multiple simulations may give better statistical results than a single longer simulation for sampled properties from MD [86,87]. However, in the case of the highly viscous DESs system, longer simulation might be necessary due to their large relaxation times [86].

Notably, when reporting the averages of protein properties from MD simulations uncertainties arising from the time-averages are not reported in many studies. Users of MD simulations are highly encouraged to properly discuss the sampling quality and quantification of uncertainties arising from MD simulations and report the calculation of their error bars in a detailed way. The usage of advanced averaging techniques for example, block averaging, which give more realistic error bars for time-averaged properties, at best calculated from different replica simulations, is encouraged. For a detailed discussion about this topic, the reader is referred to the best practice report by Grossfield et al. [86].

The main reason for the application of MD simulations is the study of biomolecules at the molecular level. They provide insights into thermodynamic and transport processes that are inaccessible through experimentation. As such, comparing the structural properties of proteins to experimentally measured protein properties, obtained from methods such as spectroscopic measurements, poses a challenge to their validation. Hence, it is crucial to validate the results from MD simulations by carefully testing the force field for the solvent mixture and/or combining the simulations with experiments of protein properties (e.g., spectroscopy measurements to obtain radii of gyration) when feasible.

## 3. Conclusions & Future Perspectives

There are different ways that DESs can affect (bio)catalysis: they can (de)stabilize the enzyme structure or affect the active site conformation, induce mass transfer limitations (mostly due to their high viscosity) or interact with the substrates/products by for example, trapping them in the DES bulk phase which lowers their reactivity. The impact of deep eutectics on the enzyme depends on the DES composition as well as on the nature of the enzyme, the reaction it should catalyze, the conditions (e.g., temperature) and a combination of these. While the presence of high concentrations of DESs could benefit the enzyme activity and stability of lipases, no such beneficial effect was found for oxidoreductases. Only minor improvements for their activity and stability could be related to small amounts of ChCl-Gly (1:2) [65,66]. However, Bittner et al. [65] suggested that this effect comes merely from the glycerol concentration and its preferential interaction with the protein surface rather than from a synergistic effect of the DES. In addition, stability of NAD(H) might be influencing these results [67]. This contrasts the findings for CALB, which can be activated only by a complex formation induced by all DES constituents making the presence of deep eutectic mixtures important [47,49].

The combination of MD simulations with experimental measurements (e.g., bulk phase properties of the solvent, protein stability, or enzymatic activity) has been shown to be essential for understanding the impact of DESs on biomolecules. MD simulations are one of the most thorough models of real mixtures and macromolecules; however, they are still a model of reality making them prone to various errors [88]. Before applying MD simulations to study novel systems, it is therefore crucial to validate the force field performance for the solvent mixture with experimental measurements (e.g., density, solvation free energy, or viscosity) or measure properties of the protein that can also be accessed by the MD simulations. Moreover, to fully understand the implications of the sampled properties from the MD simulations (such as structural properties or protein–solvent interactions), they should be compared to experimental measurements for protein stability or enzymatic activity. Only by combining experimental and computational methods can the full impact of DESs on biomolecules be captured (e.g., different RMSD/RMSF influence on lipases and ADHs discussed in Section 2.6), whereby the MD simulations can shed light on molecular processes and help to explain the experimental observations. Furthermore, gaining a comprehensive understanding of the solvent and protein’s molecular interactions can offer predictions regarding upcoming experiments and limit the experimental workload.

The diversity of biomolecular systems studied in DESs with atomistic MD simulations is unfortunately quite limited so far (see Table 1), particularly compared to the much wider variety of experimentally studied systems [6]. In the case of enzymes, the molecular modeling focusses only on two enzyme classes EC1 and EC3, and even within these classes only a few subclasses have yet been evaluated (EC1: ADH and versatile peroxidase, EC3: lipase, lysozyme, and protease). For non-enzymatic proteins, few studies regarding protein stability and crystallization have been found for a small number of proteins (recently gaining an additional two DNA structures and NAD(H)). As the DESs can influence enzymes/proteins in different ways, more atomistic molecular simulations of different enzymes in DES are needed to gain a complete picture of their interactions in these solutions.

**Figure 4:**
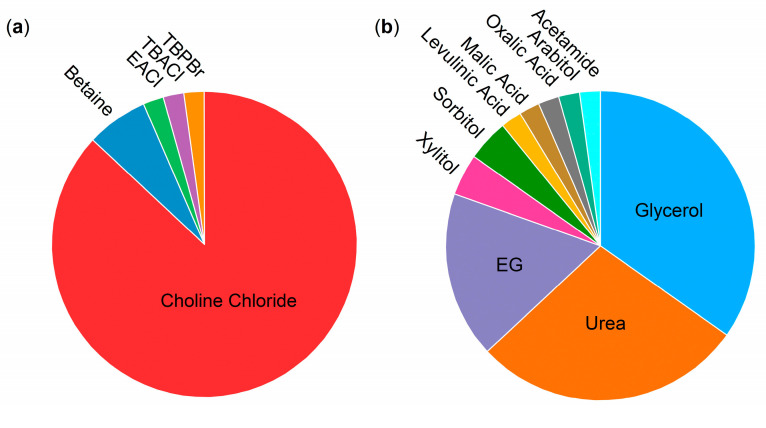
Distribution of (**a**) hydrogen bond acceptors (HBAs) and (**b**) hydrogen bond donors (HBDs) in the 46 deep eutectic mixtures reviewed within this work. Abbreviations: tetra butyl phosphonium bromide (TBPBr), tetra butyl ammonium chloride (TBACl), ethyl ammonium chloride (EACl), and ethylene glycol (EG).

Moreover, the diversity of the tested deep eutectic mixtures is mainly focused on choline chloride (87% with only a few exceptions, see Figure 4), as HBA and urea/glycerol (a combined 63%), and as HBD with some exceptions (see Figure 4). It has only recently been systematically extended to different HBA [43,49,57,65,72,73]. This is presumably due to the availability of force field parameters for the choline-based DESs for different biomolecular models, while such a systematic parametrization is lacking for many other DES components [27,28,29,89]. Rigorous force field development and validation for more (ionic) DES components and their applications for biomolecular systems are necessary for the systematic testing of DES components to fully optimize the potential of DESs for protein/enzyme applications. Thereby, Parisse et al. [60] provide a very good starting point in selecting the LJ parameters for protein/DES/water systems from different force fields that are optimized for the DES itself and its interaction with a biomolecule.

Most of the DESs systems reviewed within this work (91%) are composed of the HBA and HBD at their respective eutectic composition (see Table 1, e.g., a molar ration of 1:2 for ChCl-Urea or ChCl-Gly). However, there is no magic at this eutectic composition; it simply is the intersection of two solid–liquid equilibrium lines, one from each of the components [3,90]. Moreover, the deepness of the eutectic systems results from the properties of the liquid mixture—arising from negative deviations from an ideal solution—rather than from the solid state [3]. These negative deviations of the liquid phase are also present in non-eutectic compositions for many deep eutectic mixtures, although it might be asymmetrically distributed between the components [3]. With this in mind, the exploration of (deep) eutectic systems beyond their eutectic composition, which was exemplarily done by Sarkar et al. [70], Kovács et al. [51], and Bittner et al. [65], becomes essential. A (deep) eutectic mixture may be liquid at the desired temperature for different HBA/HBD compositions. For instance, the arbitrary deep eutectic system in Figure 1 is liquid at ambient conditions (298 K) for a mole fraction *x*_1_ from 0.4 mol/mol to 0.75 mol/mol (in other words from a ratio of 3:2 to 1:3). This makes all possible combinations of HBA/HBD within this range subject as a medium for (bio)catalysis or separation processes and adds a further degree of freedom to tailor DESs. To systematically understand and explore this expanded design space, sound thermodynamic modeling and detailed molecular simulations are necessary. For a review on the thermodynamic modeling of DESs by different models, the reader is referred to González de Castilla et al. [38].

Despite most cases not being subject to molecular simulations, the effect of DES molecules on the substrates/products of the respective interactions have been suggested to be the main contributor to different reactivity in some cases [47,49,51,77]. Therefore, it is crucial to understand the impact of the solvent mixture on the thermodynamics of the reaction and conformation of larger substrate molecules, besides its impact on the enzyme/protein structure. This thermodynamic effect of DES mixtures on the catalytic reaction equilibrium is often neglected, whereby the solvent effect on reaction equilibria has already been calculated using for example, PC-SAFT or COSMO-RS (e.g., see [91,92,93,94]).

Combining all these challenges and perspectives, further understanding of the interactions between the heterogeneous mixtures of enzymes, DES molecules (partly of ionic nature) and water can pave the way for improving (deep) eutectic mixtures in various applications, e.g., for the storage of biomolecules or as reaction medium for (bio)catalytic reactions.

## Abbreviations

DESs, deep eutectic solvents; HBA, hydrogen bond acceptor; HBD, hydrogen bond donor; ChCl, choline chloride; Gly, glycerol; MD, molecular dynamics; AMBER, Assisted Model Building with Energy Refinement; CHARMM, Chemistry at Harvard Macromolecular Mechanics; OPLS-AA, Optimized Potentials for Liquid Simulations All Atom; Δ*G*_solv_, solvation free energy; GAFF, General Amber force field; CGenFF, CHARMM general force field; CALB, *Candida antarctica* Lipase B; RMSD, root-mean-square-deviations; *R_G_*, radius of gyration; Bet, Betaine; Xyl, Xylitol; RMSF, root-mean-square-fluctuations; Ser, Serine; Asp, Aspartic Acid; His, Histidine; EG, ethylene glycol; SASA, solvent accessible surface area; LJ, Lennard–Jones; RDF, radial distribution function; OxAc, Oxalic Acid; LevAc, Levulinuc Acid; MalAc, Malic Acid; NADH, nicotinamide adenine dinucleotide; ADH, alcohol dehydrogenase; *a*_w_, thermodynamic water activity; EACl, ethyl ammonium chloride; VP, Versatile Peroxidase; UV, ultraviolet; DFT, density functional theory; TBA, thrombin binding g-quadruplex aptamer; NMR, Nuclear Magnetic Resonance; c-kit DNA, c-kit oncogene protomer g-quadruplex Deoxyribonucleic Acid; HP-36, chicken villin headpiece subdomain; BSA, bovine serum albumin; HEL4, human VH antibody fragment; TEAA, triethylammonium acetate; PC-SAFT, Perturbed Chain Statistical Associating Fluid Theory; COSMO-RS, Conductor-like Screening Model for Real Solvents.
